# The yield of immediate post lung biopsy CT in predicting iatrogenic pneumothorax

**DOI:** 10.1186/s12890-020-1128-8

**Published:** 2020-04-15

**Authors:** Rafael Y. Brzezinski, Ifat Vigiser, Irina Fomin, Lilach Israeli, Shani Shenhar-Tsarfaty, Amir Bar-Shai

**Affiliations:** 1 Department of Internal Medicine “C”, “D” and “E”, Tel Aviv Sourasky Medical Center and Sackler Faculty of Medicine, Tel Aviv University, Tel Aviv, Israel; 2 Neufeld Cardiac Research Institute, Sackler Faculty of Medicine, Tel Aviv University, Tel Aviv, Israel; 3 Tamman Cardiovascular Research Institute, Leviev Heart Center, Sheba Medical Center, Tel-Hashomer, Israel; 4 Department of Neurology, Tel Aviv Sourasky Medical Center and Sackler Faculty of Medicine, Tel Aviv University, Tel Aviv, Israel; 5 Division of Pulmonary Medicine, Barzilai Medical Center, Faculty of Health Sciences, Ben-Gurion University, 2 Hahistadrut Street, Ashkelon, Israel

**Keywords:** Biopsy, Complications, Computed tomography, Lung cancer, Pneumothorax

## Abstract

**Background:**

The most prevalent complication of percutaneous lung biopsy is pneumothorax (PNX). A routine immediate post-procedure CT scan (ICT) to spot PNX is done in many centers. However, the diagnostic yield of this practice has not been studied broadly. We sought to evaluate whether an ICT could replace the routine follow-up chest X-ray (CXR) in detecting procedure related PNX.

**Methods:**

We examined case-records of 453 patients who underwent lung biopsy at our medical center**.** We analyzed findings from CXR performed 2-h after biopsy and from CT images at the site of biopsy acquired immediately after the procedure (ICT). Multivariate analysis was used to identify the risk factors for PNX, and we examined the concordance between ICT and CXR-2-h post-procedure.

**Results:**

A total of 87 patients (19%) were diagnosed with PNX on CXR-2-h post-procedure. ICT detected 80.5% of diagnosed PNX (*p* <  0.01). However, ICT demonstrated a negative predictive value of only 94%, meaning 17 patients (6%) with a negative ICT did eventually develop PNX seen on CXR. Furthermore, bleeding surrounding the puncture area spotted on ICT negatively predicted the development of PNX (OR = 0.4 95% CI; 0.2–0.7).

**Conclusions:**

We conclude that a CT scan performed immediately after percutaneous lung biopsy cannot replace the routine follow-up CXR in predicting iatrogenic PNX. Bleeding in the needle’s tract may lower the risk for procedure-related PNX.

## Background

Computed tomography (CT) - guided percutaneous needle biopsy is a safe and precise method for the diagnosis of different pathologies in the thorax [1, 2].

The most prevalent complication of this procedure is pneumothorax (PNX), of which the reported prevalence ranges between 15 and 29% [3]. PNX ﻿can lead to a considerable reduction in the patient’s health state and longer hospitalization periods [4].

Reports regarding the risk factors and significant predictors of PNX are contradictory. A recent review highlighted lesion size, lesion depth and the presence of emphysema as significant risk factors for PNX [4], while other reports focus on the patient’s posture during the procedure [5] or the degree of emphysema seen on CT [6].

A chest X-ray (CXR) performed 1–4 h after the procedure is performed in order to diagnose biopsy-related iatrogenic PNX [7–11]. And yet, a routine immediate post-procedure CT scan (ICT) to identify PNX is also done in many centers. The question of whether an ICT could replace the routine CXR performed during follow-up in diagnosing iatrogenic PNX has not been studied in a large sample of patients.

Therefore, we sought to examine the diagnostic yield of ICT in identifying iatrogenic PNX diagnosed on the follow-up CXR after lung biopsy. We aimed to evaluate the specific risk factors for PNX present in our cohort and to determine the characteristics of patients with undetected PNX on ICT that was detected only on CXR. The ultimate goal was to assess whether an ICT can replace the follow-up CXR in patients undergoing percutaneous lung biopsy.

## Methods

### Study design

We retrospectively examined case-records of 453 patients (mean age 69 yr, 55% male) who underwent percutaneous lung biopsy in the Department of Pulmonary Diseases at our medical center**.** We reviewed the patients’ imaging studies (chest CT results acquired immediately after the procedure (referred to as ICT) and CXR performed 2-h after biopsy). We collected demographic data as well as relevant medical history such as smoking history and the presence of emphysema.

The study was approved by the local ethics committee, conformed to the principles outlined in the Declaration of Helsinki, and Informed consent was waived due to the retrospective nature of the study.

### Percutaneous lung biopsy and image analysis

All patients underwent CT-guided percutaneous lung biopsy in accordance with common procedure guidelines [10, 11]. We performed core biopsies with an 18-gauge needle without the use of a coaxial needle. Patients did not receive any conscious sedation during the procedure. We categorized patients according to the performing physician. A total of 7 pulmonary specialists and 1 chest radiology specialist participated in our study. All performing physicians had at least 3 years of experience in performing lung biopsies. The number of punctures was documented for each technique as wells as the patient’s posture during the procedure (i.e. decubitus, supine and prone). Biopsy samples were examined by a certified pathologist and were defined as malignant/benign/non-diagnostic.

An ICT was done immediately after the removal of the needle in order to document potential immediate complications such as PNX or bleeding. The ICT included CT images at the site of the biopsy. The patients were observed for 2-h at the vicinity of the procedure room. CXR was performed if any clinical deterioration was present or after completion of the 2-h observation period. Any clinical complication was treated immediately at the procedure site.

ICT scans and CXRs underwent manual surveys by trained pulmonologists at our medical center. We measured the lesions’ size in both length and width, their location and distance from pleura, and documented the presence of bleeding surrounding the puncture area. A lesion’s location was defined as being present at the upper lobes (both left and right) vs. lower lobes (including right middle lobe).

### PNX diagnosis

In our institution, the follow-up CXR is routinely obtained 2 h after biopsy in order to assess procedure-related PNX. Patients were categorized into two groups according to the presence of PNX on CXR. The need for chest tube insertion was documented for all patients diagnosed with PNX.

### Statistical analysis

All continuous variables are displayed as mean ± SD for normally distributed variables or median [interquartile range] for variables with abnormal distribution. Categorical variables are displayed as numbers (%) of subjects within each group. The different characteristics in patients diagnosed with and without PNX were compared by a Student’s *t*-test for normally distributed variables and by the Mann-Whitney *U* test for non-normally distributed ones. To assess associations among categorical variables, we used a chi-square test. We measured sensitivity and specificity values for the ability of ICT to detect PNX present on CXR 2-h after the procedure.

To isolate significant risk factors for the development of PNX, we performed multivariate logistic regression to predict the presence of PNX on CXR. The model was adjusted for the following covariates: age, gender, smoking status, number of pack-years, lesion length and width, lesion distance from the pleura, categorized location of the lesion, performing physician, number of punctures performed, patient posture, pathologic diagnosis and the presence of bleeding on ICT. The variables included in our multivariate analysis were the ones that demonstrated statistical significance on univariate analysis, along with additional risk factors for iatrogenic PNX highlighted in past reports [4–6].

We performed one-way Analysis of Variance (ANOVA) with Tukey’s test for multiple comparisons to compare characteristics between the four different groups of patients according to ICT results; false/true negative and false/true positive test results. *P*-values of < 0.05 were considered statistically significant.

We used the IBM SPSS Statistics 22.0 statistical package (IBM Corporation, Armonk, New York, USA) and GraphPad Prism version 7.00 (GraphPad Software, La Jolla, CA, USA) for all statistical analysis.

## Results

### Study population

A total of 87 patients (19%) were diagnosed with PNX on CXR performed 2-h post-procedure. Twenty-five patients required chest tube insertion accounting for 29% of PNXs and 5.5% of all lung-biopsies. Patients’ characteristics are shown in Table 1. Patients diagnosed with PNX presented with smaller lesions in both length and width, as well as a more distant location from the pleura (Table 1). Notably, patients with and without PNX demonstrated a similar prevalence of existing co-morbidities such as emphysema and smoking history. Men had slightly higher rates of PNX compared to women, albeit not statistically significant; 61% vs 54%, (*p* = 0.15). The number of needle passes during the procedure was slightly higher in patients without PNX; 1.7 ± 0.6 vs. 1.5 ± 0.6, (*p* = 0.02).

**Table 1:** Patient-Characteristics According to Diagnosed Pneumothorax on Chest X-ray 2-h Post-Lung Biopsy

Characteristic	Negative CXR	PNX on CXR
Number of subjects	366 (81%)	87 (19%)
Age, years	70 ± 13	68 ± 13
Gender (Male)	198 (54%)	53 (61%)
History of Smoking	220 (60%)	42 (48%)
Pack Years	17 ± 30	25 ± 32
Emphysema Diagnosis	30 (8%)	8 (9%)
Lesion length, cm	2.9 ± 1.9	2.1 ± 1.4
Lesion width, cm	2.5 ± 2	1.8 ± 1.2
Distance from pleura, cm	1.7 ± 3.4	2.7 ± 1.8
Lesion Locus		
Upper Lobes	158 (43%)	43 (49%)
Lower Lobes	209 (57%)	44 (51%)
Number of punctures performed	1.7 ± 0.6	1.5 ± 0.6
Patient Posture		
Prone	221 (60%)	54 (62%
Supine	118 (32%)	21 (24%)
Decubitus	27 (8%)	12 (14%)
Bleeding observed	236 (64%)	53 (60%)
Pathologic Diagnosis		
Non-Diagnostic	19 (5%)	7 (8%)
Benign	131 (36%)	39 (45%)
Malignant	217 (59%)	41 (47%)

Data presented as mean ± SD or N (%). *CXR- Chest X-ray*; *PNX- Pneumothorax*

### Detected bleeding on ICT lowered the risk for PNX

**Fig. 1:**
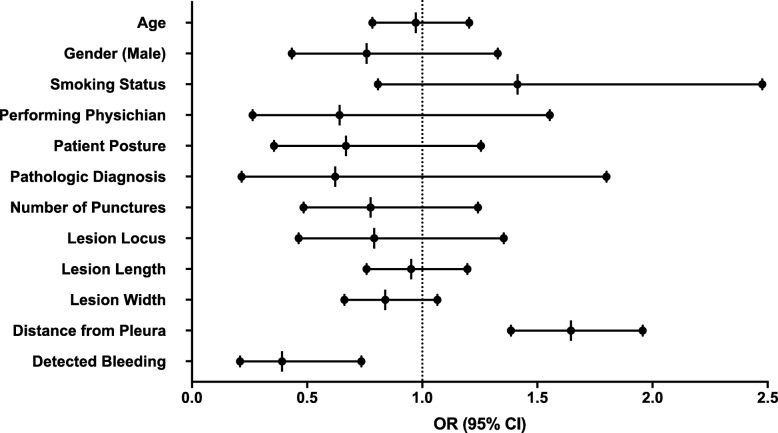
Multivariate Analysis to Predict Pneumothorax Diagnosis on Chest X-ray. The forest plot presents the odds ratio of multivariate logistic regression to predict pneumothorax (PNX) on chest X-ray (CXR) 2-h post-lung biopsy. The sampled lesion’s distance from the pleura and detected bleeding on immediate post-procedure CT are the only significant predictors in the model. Deeper lesions predict higher rates of PNX (*p* < 0.001) and detected bleeding seems to have a protective effect (*p* = 0.004). *OR - Odds Ratio; CI- Confidence Interval*

We first sought to evaluate the risk factors for developing PNX in our cohort (Fig. 1). In-line with previous reports [3, 4], multivariate analysis showed that the lesion’s distance from the pleura predicted higher rates of PNX (OR = 1.6, 95% CI; 1.4–1.9). However, the lesion’s size, categorized location, number of needle passes, and the patient’s posture during the procedure were not significant predictors of PNX in our cohort (Fig. 1). The pathologic diagnosis and the performing physician were not significant predictors as well.

Finally, our analysis demonstrated that bleeding surrounding the puncture area spotted on ICT negatively predicted the development of PNX on CXR (OR = 0.4 95% CI; 0.2–0.7, *p* = 0.004) (Fig. 1).

### ICT did not detect all PNX seen on CXR

**Fig. 2:**
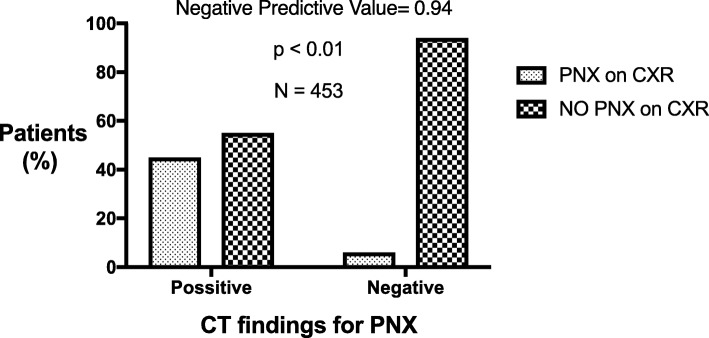
The Yield of Post-Lung Biopsy CT in Predicting Pneumothorax on Chest X-Ray 2-h Post-Procedure. The clustered bar chart shows the percentage of patients diagnosed with pneumothorax (PNX) on Chest X-ray 2-h post-procedure within patients diagnosed with (‘Positive’) and without (‘Negative’) PNX on immediate CT. *P*-value was calculated by a chi-square test. *CT- computed tomography; PNX- Pneumothorax*

We next sought to determine the diagnostic yield of ICT in detecting iatrogenic PNX. ICT detected 80.5%, of patients diagnosed with PNX on CXR (*p* <  0.01). More importantly, ICT demonstrated a negative predictive value of only 94%, meaning 17 patients (6%) with a negative ICT did eventually develop PNX seen on CXR 2-h post-procedure (Fig. 2).

ICT identified 85 patients (23%) with PNX that did not develop into a significant PNX identified on CXR, accounting for a specificity rate of 77% for ICT in predicting PNX.

A total of 22 patients that had PNX spotted on ICT required chest tube drainage (14.2% of positive ICT patients). Three more patients with a negative ICT (1% of negative ICT patients) also required a chest tube insertion.

### Miss-diagnosed PNX on ICT related to smaller and more distant lesions

**Fig. 3:**
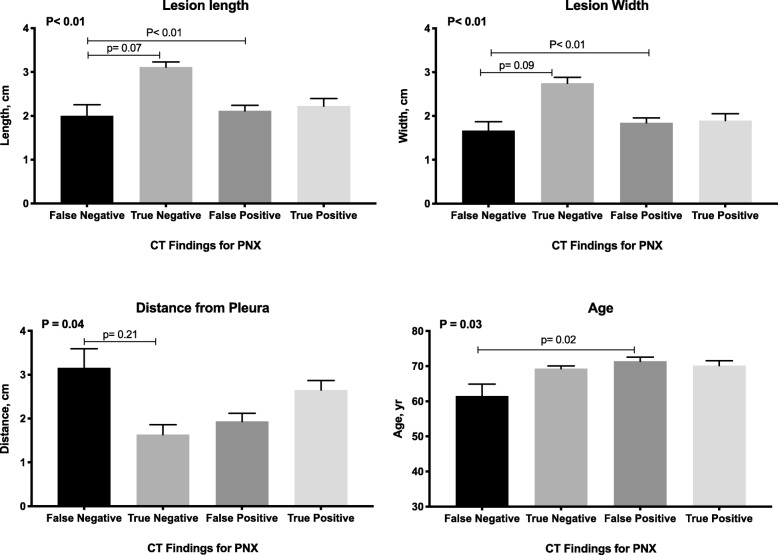
Characteristics of Lesions According to Post Lung Biopsy CT results. We categorized the cohort according to test results of the immediate post lung biopsy CT in predicting pneumothorax on chest X-ray. Patients with a false negative test result had smaller lesions in both length (upper left), width (upper right), were deeper within the lung (lower left) and younger in age (lower right). Data presented as mean ± SEM. *P*-values were calculated by one-way ANOVA followed by Tukey’s test for multiple comparisons. *CT- Computed Tomography; PNX- Pneumothorax*

Finally, we aimed to characterize the sub-group of patients (6%) that had a diagnosed PNX on CXR that was not detected on ICT (Table 2). These patients presented with smaller lesions in both length and width, as well as a more distant location from the pleura (Fig. 3). Of note, this sub-group of patients were relatively younger than patients with PNX spotted on both ICT and CXR; 61 ± 14 vs. 69 ± 13 (*p* = 0.02) (Table 2, Fig. 3).

**Table 2:** Characteristics of Patients with a Negative CT Post- Lung Biopsy According to Diagnosed Pneumothorax on Follow-up Chest X-ray

Characteristic	No PNX on CXR	PNX on CXR	***P***-value
Number of subjects	282	17	
Age, years	**69 ± 13**	**61 ± 14**	**0.02**
Gender (Male)	150 (53%)	10 (59%)	0.8
History of Smoking	113 (40%)	6 (35.3%)	0.8
Pack Years	18 ± 31	17 ± 28	0.86
Emphysema Diagnosis	20 (7%)	0	0.6
Lesion length, cm	**3.1 ± 2.1**	**2 ± 1.1**	**< 0.01**
Lesion width, cm	**2.8 ± 2.2**	**1.6 ± 0.8**	**< 0.01**
Distance from pleura, cm	**1.6 ± 3.8**	**3.2 ± 1.8**	**< 0.01**
Lesion Locus			
Upper Lobes	117 (42%)	11 (65%)	0.06
Lower Lobes	165 (58%)	6 (35%)	
Number of punctures performed	1.8 ± 0.7	1.5 ± 0.5	0.16
Patient Posture			
Prone	**168 (60%)**	**11 (65%)**	**0.01**
Supine	**96 (34%)**	**2 (12%)**	
Decubitus	**18 (6%)**	**4 (23%)**	
On-spot Pathologic Diagnosis			
Non-Diagnostic	15 (5%)	3 (18%)	0.11
Benign	102 (36%)	6 (35%)	
Malignant	165 (58%)	8 (47%)	

*P*-values < 0.05 are shown in bold

Data presented as mean ± SD or N (%). *CXR- Chest X-ray*; *PNX- Pneumothorax*

## Discussion

The main finding of this study is that an ICT cannot replace a CXR performed 2-h post-lung biopsy in detecting iatrogenic PNX. In this single-center experience, ICT detected 80% of PNX seen on the follow-up CXR with a 6% rate of false negative test results. Approximately 1% of patients with a negative ICT ended up requiring a chest tube insertion. PNX is a life-threatening complication and this diagnostic yield does not support waiving the follow-up CXR in detecting PNX.

Our findings are in-line with a recent report by Lim et al. [12] that depicted the time-dependent incidence of PNX after lung biopsy. Similar to our findings, an ICT detected 81% of PNX seen on CXR 4-h post procedure. Lim et al. reported an even higher rate of false negative results (~ 18%) and this might be due to the longer follow-up period in their study (4 vs 2 h post procedure).

The incidence of PNX in our cohort (19%) is similar to previous reports regarding lung biopsy complication rates recently reviewed [3]. The significant risk factors for this complication vary across different study cohorts [4–6, 8, 13]. Our multivariate analysis shows that the distance of the sampled lesion from the pleura is the main risk factor for PNX. This is in accordance with the majority of past reports [3, 4, 11]. However, lesion size or patient posture were not significant risk factors in our cohort.

Our findings show, for the first time, that detected bleeding on ICT was protective against the development of PNX (OR = 0.4 95% CI; 0.2–0.7, *p* = 0.004). This concept has been discussed previously (personal communication) but did not have sufficient supporting evidence until now. We hypothesize that processes associated with blood clot formation at the puncture-area affect the visceral pleura and might aid in preventing air leak from the lung parenchyma to the pleural space. Future more mechanistic-oriented studies aimed to test this theory are warranted. Lim et al. reported a similar protective effect for the presence of hemoptysis in the development of PNX (HR = 0.503; CI = 0.355–0.713) [12]. Detected bleeding on ICT is seen earlier and could, in some cases, lead to hemoptysis on follow-up. Their findings provide further support for our theory.

Our study has several limiting aspects. Primarily, this is a single center retrospective experience and is hence subject to residual confounding effects. And yet, our major complication rates are in-line with previous reports and thus provide support to the external validity of our findings. Furthermore, this study was not designed to include the effect of final biopsy results nor the diagnostic yield of the lung biopsy itself. However, the pathologic results did not show significant correlations between the type of sampled lesion and the risk for PNX. Finally, future studies should include a quantitative assessment of the size of spotted PNXs in both CXR and ICT.

The future clinical implication of our findings is debatable. Our study demonstrates the limited yield of ICT in predicting one of the major complications of percutaneous lung biopsy. In light of the immediate high risk of PNX in this clinical scenario, it does not seem possible to perform an ICT without also obtaining a CXR 2-h after the procedure. However, completely avoiding ICT is also inconceivable since ICT aids to detect other major and life-threatening complications (e.g. massive bleeding and tension PNX). Future prospective studies are warranted to better determine the ultimate time point to perform the first follow-up CXR after lung biopsy.

## Conclusions

A CT scan performed immediately after lung biopsy cannot replace a routine chest X-ray 2-h post-procedure in assessing iatrogenic pneumothorax. Bleeding in the needle’s tract may lower the risk for procedure-related pneumothorax.

## Data Availability

The datasets used and/or analyzed during the current study are available from the corresponding author on reasonable request.

## Abbreviations

CT: Computed tomography
CXR: Chest X-ray
ICT: Immediate post-procedure CT scan
PNX: Pneumothorax
